# 2481. Interactive Dashboards for Improved Tracking and Communication of CLABSI Prevention Efforts

**DOI:** 10.1093/ofid/ofad500.2099

**Published:** 2023-11-27

**Authors:** Guillermo Rodriguez-Nava, John Shepard, Karen McIntyre, Lucy S Tompkins, Jorge Salinas

**Affiliations:** Stanford University School of Medicine, Palo Alto, California; Stanford University, Palo Alto, California; Stanford Healthcare, Stanford, California; Stanford University, Palo Alto, California; Stanford University, Palo Alto, California

## Abstract

**Background:**

Central line-associated bloodstream infections (CLABSI) are associated with increased morbidity, mortality, and healthcare costs. Many CLABSIs can be prevented using evidence-based care. To make this information more accessible and actionable, we developed interactive dashboards that translate data on CLABSI-related metrics and prevention efforts into visual formats that can be easily understood by healthcare professionals.

**Methods:**

A multidisciplinary work group of data analysts, infectious diseases physicians, and infection preventionists determined the content and layout of the dashboards. A query was written to extract necessary data elements from the Electronic Medical Record and NHSN. Data was then exported and used to build the dashboards using Tableau® software. The dashboard can be filtered at the facility level or at the unit level.

**Results:**

We present the dashboard of one general ward **(Figure 1)**, one intensive care unit (ICU) **(Figure 2)**, and one cardiovascular ICU (CVICU) **(Figure 3)**. The central line standard utilization ratio (SUR) was higher for the CVICU ( >1.5), followed by the ICU (≈1), and the general ward (< 1). Blood culture intensity (number of blood cultures collected/patient-days) was less for the general ward (< 5%), variable for the ICU (8-14%), and persistently higher for the CVICU (11%). Compliance with daily chlorhexidine bathing was higher for both the CVICU (80%) and the ICU (75%) compared to the general ward (40-60%). The CLABSI rate per 1,000 central line-days has been downtrending for the CVICU and uptrending for the ICU. Besides lower compliance with daily chlorhexidine bathing, the examined ICU has also recently shown a higher proportion of long-term devices (40%), calculated as proportion of central lines ≥ 7 days/all central lines, compared to the CVICU (25%).

**Figure:**
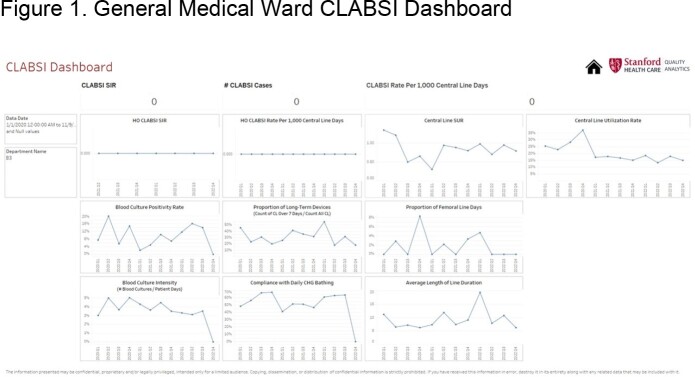

**Figure:**
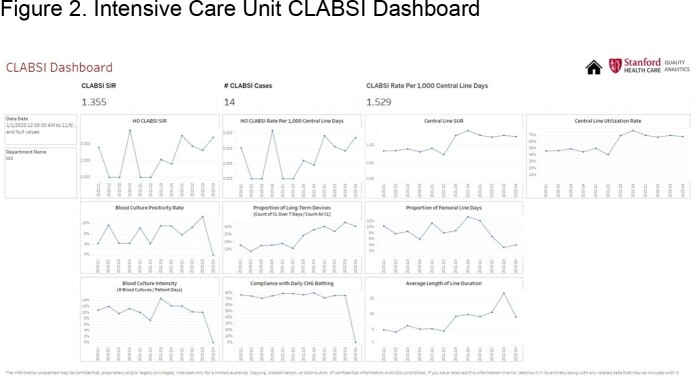

**Figure:**
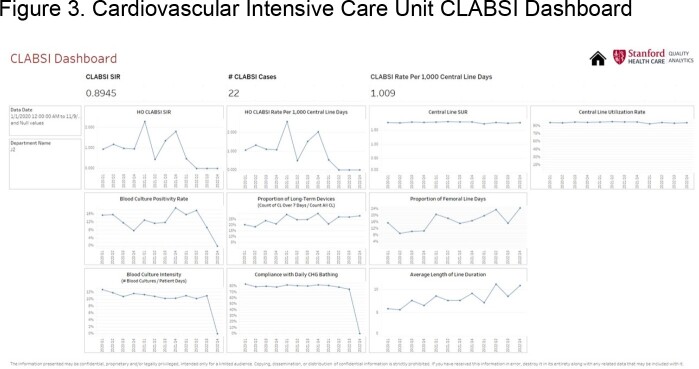

**Conclusion:**

Dashboards should contain meaningful outcomes and standardized process metrics that are mapped to strategic goals and be timely to support prompt identification of deviations. Our CLABSI dashboards are effective tools for communicating and tracking performance data. Filtering at the unit level allows us to identify unique unit characteristics, or “fingerprints,” and recognize specific areas for improvement. This enables us to develop targeted interventions for each unit.

**Disclosures:**

**All Authors**: No reported disclosures

